# Understanding Emotion Inflexibility in Risk for Affective Disease: Integrating Current Research and Finding a Path Forward

**DOI:** 10.3389/fpsyg.2019.00392

**Published:** 2019-02-27

**Authors:** Karin G. Coifman, Christopher B. Summers

**Affiliations:** Kent State University, Kent, OH, United States

**Keywords:** emotion, affect, flexibility, disorder, anxiety, depression

## Abstract

Emotion-related disorders (e.g., depression, anxiety, stress, eating, substance and some personality disorders) include some of the most common, burdensome, and costly diseases worldwide. Central to many, if not all of these disorders, may be patterns of rigid or inflexible emotion responses. Indeed, theorists point to emotion in-flexibility as a potential cause or maintaining factor in emotion-related diseases. Despite the increasing prominence of emotion inflexibility in theories of affective disease, a comprehensive review of the developing empirical literature has not yet been conducted. Accordingly, this review will examine the three dominant lines of inquiry assessing emotion flexibility. These include: (1) the capacity to use and vary deliberate emotion regulation strategies, (2) the context sensitivity of spontaneous emotional responses, and (3) flexibility in the appraisal of emotional events and experiences. Moreover, current evidence suggests that each of these three lines of research may converge to suggest the interplay of two key biological dimensions in emotion inflexibility, threat sensitivity, and cognitive control, known to be impaired in patients with affective disorders. In short, this developing body of work suggests a path by which future research could explicate and even exploit the ties between emotion inflexibility and affective disease, contributing to the development of improved models of risk, assessment, and intervention, with broad implications for psychological health.

## Understanding Emotion Inflexibility in Risk for Affective Disease: Integrating Current Research and Finding a Path Forward

The prevalence of emotion-related psychiatric disorders has reached nearly epidemic proportions (Kazdin, 2007). Rates for the more common anxiety, stress, and depressive disorders suggest that the average adult can have a projected lifetime risk of up to 50% (Kessler et al., 2012). Overwhelming evidence indicates that most disorders emerge at the intersection of pre-existing vulnerability (genetic, learned) and significant, stressful, and emotion-laden life events. However, the ability to reliably model risk for disease, even after acute, stressful events, remains elusive. Current models of emotion-related risk do not adequately account for this confluence of biological and situational factors. Indeed, given the significant burden of common affective disorders (depression, anxiety, stress disorders) on society and the individual, how these factors come together to inform emotion-related risk versus psychological health is a critical public health issue. Hence, identifying patterns of risk-related emotion processing is a key step in improving the reliability of assessments of risk and the efficacy of early intervention. Accordingly, in this review, we will focus on characterizing the role of in-flexible emotion responding in the development and/or maintenance of affective disease. This burgeoning field of research is well supported by theories of affective disease and health but has not been subject to review as yet. Hence, here, we examine the three dominant lines of research linking emotion in-flexibility with affective disease. Moreover, we discuss the likely biological dimensions underlying emotion in-flexibility and propose a future research agenda that applies this emotion processing framework to further the development of risk assessment and intervention for those most in need. Given the nascency of emotion flexibility as a construct, this review errs on the side of inclusivity, exploring relevant research under the umbrella of emotion flexibility, albeit, often by various names (e.g., rigidity, flexibility, regulatory variability).

## Emotion Flexibility and Emotional Health

Emotion flexibility is increasingly viewed as a cornerstone of psychological health. Theorists largely agree that emotion flexibility encompasses automatic, implicit, as well as deliberate emotion regulatory processing and is defined as the ability to respond to shifting demands with contextually sensitive modulation of emotional responses (Kashdan and Rottenberg, 2010; Waugh et al., 2011; Bonanno and Burton, 2013; Ottaviani et al., 2013; Aldao et al., 2015; Hollenstein, 2015; Coifman and Almahmoud, 2016). Indeed, emotion flexibility could be considered a superordinate construct such that it includes the ability to generate or up-regulate emotion, as well as the ability to inhibit or down-regulate emotion, facilitating adaptation to challenges as well as routine or daily functioning. Emotion flexibility may be particularly well suited to help inform models of emotion-related risk as it appears to characterize the optimal balance of “bottom-up” threat-related processing and “top-down” cognitive control (Dennis and Chen, 2007; Ochsner and Gross, 2007; Coifman et al., 2018). These biologically based, constituent dimensions underlie all emotion processing and both dimensions are broadly implicated in affective disorders (Insel et al., 2010). In particular, there is evidence that both dimensions are heritable (Hariri and Holmes, 2006; Engelhardt et al., 2015; Gustavson et al., 2015) and the interplay between them may shift through environmental input and/or learning resulting in the development of clinical impairment (Venables et al., 2015; Mather et al., 2016; Nelson et al., 2016). The imbalance of the constituent dimensions of emotion flexibility may influence symptom development including common trans-diagnostic phenomena, such as disruptions in sleep (e.g., Zoccola et al., 2009), negative repetitive thought or rumination, persistent negative mood, and even behaviors associated with disease maintenance (e.g., social avoidance, Kimbrel, 2008; Trull et al., 2015).

A key element of emotion flexibility is the ability to inhibit or down-regulate emotional responses. Indeed, poor emotion inhibitory processing is well recognized as a significant predictor of the most prevalent affective disorders, including depression (Gotlib and Joormann, 2010), post-traumatic stress disorder (Charney et al., 1993; Cohen et al., 2013), anxiety disorders (Mathews and MacLeod, 2005), and even bipolar disorders (Gruber, 2011). Moreover, current evidence suggests that this same pattern of emotion in-flexibility can reliably predict the onset of pathology following a stressful life event (Coifman and Bonanno, 2010) as well as significant symptomatology (Moran et al., 2012), disease course, and responsiveness to treatment (Rottenberg et al., 2002, 2005). Accordingly, emotion in-flexibility could be a phenotype for affective disease, evident across clinical samples, and highly predictive of the onset and course of affective disorders.

In contrast, research and theory argue convincingly for a link between emotion flexibility and adaptation. For example, most contemporary models of emotion posit that emotions evolved to serve discrete functions in order to facilitate survival and adaptation to specific environmental threats and demands. Accordingly, there is clear evidence for discrete functions for emotions such as fear (Ohman and Mineka, 2001), sadness (Bonanno et al., 2008), anger (Lerner and Keltner, 2001), disgust (Tybur et al., 2013), joy, and happiness (Gruber et al., 2011) to facilitate survival as well as social living. Importantly, implicit in theory is the notion that emotions are brief episodes, only adaptive within the context for which they evolved. For example, fear is highly adaptive in the presence of a true threat (c.f. Ohman and Mineka, 2001) as it is associated with physiological changes that facilitate efficient responses to threat for the individual (e.g., increases in sympathetic autonomic activity) as well as behavioral signals that are adaptive for the larger group. However, when fear extends beyond the context of a true threat, it is maladaptive, and patterns of contextually insensitive or over-generalized fear responses are consistently tied to poor psychological functioning and increased risk of psychiatric disease (Buss et al., 2004; Graham and Milad, 2011; Craske et al., 2012). Similar evidence exists for most other emotions, including even joy (Gruber et al., 2011). Indeed, embedded in dominant models of emotion, emotion regulation, and affective disorders is the notion that emotions are adaptive yet contextually bound (Ekman, 1992; Cole et al., 1994; John and Gross, 2004; Kring, 2008; Nolen-Hoeksema and Watkins, 2011). Hence, adaptive emotional processing must include frequent and flexible modulation as circumstances and demands shift. As such, much emotion processing likely proceeds implicitly or automatically, so as to not deter from more important activities that require deliberate or conscious action (Koole and Rothermund, 2011). Indeed, we can define emotion flexibility as the ability to respond to shifting emotional contexts, including environmental contexts as well as internally elicited emotion (c.f. Ochsner et al., 2009) with appropriate modulation of emotional responses, encompassing automatic, implicit as well as deliberate processing. This includes the ability to generate or up-regulate emotions in response to contextual factors as well as the ability to shift or down-regulate emotions as contextual parameters or features change (Kashdan and Rottenberg, 2010; Waugh et al., 2011; Bonanno and Burton, 2013; Aldao et al., 2015; Hollenstein, 2015; Coifman and Almahmoud, 2016).

While emotional flexibility shares some conceptual overlap with the broader construct of psychological flexibility, it also is distinct from psychological flexibility and incrementally informative toward our understanding of psychological health and dysfunction. Psychological flexibility is broadly defined as the ability to modulate cognitive and behavioral actions in pursuit of a goal across shifting contexts (Kashdan and Rottenberg, 2010; Morris and Mansell, 2018). Research suggests that psychological flexibility is generally associated with favorable outcomes, such as well-being and life-satisfaction (Graham et al., 2016; Wersebe et al., 2018). Psychological rigidity on the other hand, is characterized by stagnant, cognitive-behavioral patterns impervious to contextual feedback, despite potentially adverse consequences (Morris and Mansell, 2018). Such rigidity permeates the affective disorders (Levin et al., 2014). One key difference between emotional and psychological flexibility lies at the level of analysis, with emotional flexibility providing more granular insight into how one’s affective repertoire contributes to their overall psychological flexibility. This micro-level of analysis may provide unique insights that its macro counterpart may not be able to offer. For example, emotional flexibility may provide a clearer understanding of how affective regulation relates to goal-pursuit, in the broader context of psychological flexibility. Indeed, not only are emotions tightly linked to behaviors in the service of achieving goals, but goal-pursuit also comprises an essential component of psychological flexibility (Kashdan and Rottenberg, 2010). Thus, refining our understanding of emotional flexibility serves as both a unique and an essential complement toward the broader understanding of psychological flexibility.

## Emotion In-Flexibility and Risk for Affective Disease

Research on emotion in-flexibility and disease has proceeded along three primary lines of inquiry. Much of the work has occurred within the context of stressful life events and the potential for new onset or resurgence of symptoms. However, some research has focused explicitly on clinical groups diagnosed with affective disorders. In particular, researchers aiming to capture emotion flexibility have relied heavily on lab paradigms or sampling that can capture variability within person rather than relying simply on between-person variability. Indeed the study of emotion flexibility demands repeated assessment of the individual across contexts, circumstances, and/or time, so as to provide ample opportunity to capture changing responses, whether directed deliberately or spontaneously emerging.

### Flexibility in Use of Emotion Regulatory Strategies

Researchers have defined emotion flexibility as the capacity to vary the use of deliberate emotion regulation strategies. Based largely on recent elaboration of dominant models of emotion regulation (Aldao and Nolen-Hoeksema, 2012; Opitz et al., 2012; Bonanno and Burton, 2013; Gross, 2015) researchers have increasingly argued that flexible engagement in deliberate emotion regulatory action is highly adaptive and associated with psychological health. This includes having a variable repertoire of strategies, including but not limited to reappraisal, suppression, distraction, reflection, and support seeking, that are flexibly applied when contextual parameters and individual needs demand (Sheppes, 2014). Implicit in this argument is the notion that rigid reliance on particular strategies is associated with affective dysfunction and risk for disease. Indeed, for several decades, evidence has accumulated suggesting that individuals with affective disorders typically report heavy reliance on less-adaptive strategies such as rumination, avoidance, and expressive-suppression on trait measures of emotion regulation (Dennis, 2007; Aldao et al., 2010; Koval et al., 2012; Kashdan et al., 2013; Smith et al., 2018). However, there is a troubling lack of concordance between trait and state measurement of emotion regulation (e.g., Brockman et al., 2017). Indeed, research focused on indexing flexibility in emotion regulatory processing has relied heavily on non-trait measurement, emphasizing experience sampling paradigms (benefitting from increased ecological validity and reduced risk of memory bias) and/or lab-based performance paradigms (i.e., measuring the regulatory behaviors as they are selected at repeat instances or participant success at strategy use across contexts).

In particular, emotion regulatory flexibility has been indexed in lab using two primary paradigm types. The first, developed by Sheppes and colleagues, is typically characterized as a “regulatory-choice” paradigm, eliciting high- and low-intense negative emotions *via* static images and asking participants to select a regulatory strategy (often one of two) including, for example, an option like reappraisal, which forces engagement with the emotional content, or alternatively, distraction, which facilitates disengagement from emotion material (Sheppes et al., 2014). This paradigm has been used convincingly over several studies to demonstrate that psychologically healthy individuals typically exhibit flexibility in their selection patterns, opting for more engagement-related strategies, like reappraisal, when negative emotional intensity is low, but selecting disengagement-related strategies, like distraction, when negative emotion intensity is high (Sheppes et al., 2011; Shafir et al., 2015). Indeed, recent work examining firefighters at high risk for PTSD demonstrated that regulatory-choice flexibility moderated the association between traumatic-event exposure and PTSD symptomatology such that individuals exhibiting greater flexibility were protected from risk associated with increased trauma exposure (Levy-Gigi et al., 2016).

Another prominent lab-based paradigm assesses emotion regulatory flexibility as responsiveness to regulatory directions and relies heavily on performance indices of emotion regulatory action (e.g., facial behavior). Bonanno and colleagues developed this within-subject paradigm in which individuals are directed to either visibly suppress or express emotional expression in response to evocative pictures. A higher combined score capturing both behaviors (within-subject deviations scored *via* coded facial emotion behavior) is conceptualized as flexibility. This paradigm has been influential, demonstrating the clear association between emotion regulatory flexibility and psychological health (Gupta and Bonanno, 2011) as well as flexibility and psychological resilience following trauma (e.g., 9/11: Bonanno et al., 2004). Moreover, emotion regulatory flexibility as indexed by this paradigm has demonstrated relative stability in its association to adjustment over years (Westphal et al., 2010). Finally, there is new evidence suggesting clear deficits in emotion regulation flexibility, per this paradigm, in patients diagnosed with affective disorders. For example, Rodin and colleagues examined performance on this task in veterans diagnosed with affective disorders, including some with PTSD and/or depression, and found evidence of deficits in expression enhancement linked to PTSD and depression whereas no difference by diagnosis in expression suppression (Rodin et al., 2017).

Alternatively, researchers have investigated natural or spontaneous variability and utility of emotion regulatory strategy use in daily life *via* experience sampling as well as in some lab paradigms. Indeed, across a number of investigations it is increasingly clear that individuals generally report that they rely on multiple strategies in daily life, sometimes at one point in time, with astonishing variability (e.g., Brockman et al., 2017; Kalokerinos et al., 2017; Eldesouky and English, 2018). Moreover, it is also clear that specific strategies are variable in their perceived (self-reported) effectiveness as well as on objective indices of affective change pre- and post-strategy reports both *via* experience sampling (e.g., Heiy and Cheavens, 2014) and in lab (Gruber et al., 2012). Interestingly, variability in reports and the effectiveness of particular strategies do not appear to be substantially impacted by psychological health in experience sampling but do differ in clinical versus non-clinical samples in lab. For example, Brans and colleagues examined the utility and variability of six emotion regulatory strategies across two samples, one of which exhibited the full range of depressive symptoms. The effectiveness of the six strategies at reducing subsequent negative affect was similarly limited across both samples as well as the variability in reported strategy use (Brans et al., 2013). In contrast, Gruber and colleagues examined spontaneous report of emotion regulatory strategies during a series of evocative films in lab. Patients with bipolar disorder reported greater strategy use relative to healthy control participants, but also reported less effectiveness (Gruber et al., 2012).

Although research on flexibility in emotion regulatory strategy use and selection is increasing at a rapid pace, there are meaningful limitations that warrant explicit discussion across the spectrum of this line of research. First, focus on deliberate emotion regulatory strategies is inherently limiting as it only targets what is a small minority of emotion regulatory action. Indeed, it is increasingly noted in affective science that a large proportion of emotion regulatory processing manifests implicitly and/or automatically and is largely outside of awareness (e.g., Bargh and Williams, 2007; Mauss et al., 2007; Koole and Rothermund, 2011). Hence, whether research is naturalistically sampling reports of strategy use in daily life or testing the ability to engage in deliberate strategies in lab, it, by definition, artificially limits the spectrum of emotion regulatory action that can be understood. Indeed, this challenge is quite pressing as currently the vast majority of research on emotion regulatory processing has been limited to reports of strategy use by individuals, despite the strikingly weak concordance even across trait and state measurement of the same constructs (Brockman et al., 2017). It remains an open question as to how to best integrate emotion regulatory strategy use/selection into broader models of emotion processing, and likely other non-strategy-oriented research must be bolstered and combined with strategy research so as to better explicate the overall impact of deliberate regulatory strategies, and related flexibility, in all emotion regulatory action.

### Flexibility in Spontaneous Emotional Output

Rather than focusing on particular regulatory strategies, other research on emotion flexibility has focused on the responsivity of spontaneous emotion output to shifting, emotionally evocative contexts. The primary benefit of this approach is that it is inclusive of deliberate, automatic, and implicit regulatory responses, as participants are given no specific regulatory instructions. Moreover, these paradigms typically measure and analyze emotional output on multiple dimensions by context, relying heavily on objective indicators of emotion such as coded facial behavior, autonomic activity or emotion-modulated startle. Indeed, responsive shifts of emotion output that correspond to changing contextual demands are considered evidence of flexible and adaptive emotion regulation (c.f., Coifman and Bonanno, 2009; e.g., Waugh et al., 2011). As such, adaptive responses are those that match contextual demands, or are “context-sensitive” whereas maladaptive responses are mismatched to the context, or “context-insensitive.” For example, in an interview paradigm with a fixed sequence of evocative questions, Coifman and Bonanno (2010) demonstrated that negative emotions still present during an explicitly positive context; in this case, a prompt to discuss a recent positive event that followed a prompt to discuss a recent negative event constituted inflexible negative emotion or poor emotion-context sensitivity as scores predicted elevated depression 18 months following the loss of a loved one. Indeed, this pattern of sustained negative emotional responding has been found in several studies on emotion in depressed patients (e.g., Siegle et al., 2001, 2002; Rottenberg et al., 2002) as well as repeatedly in both cross-sectional and longitudinal investigations of anxiety (Buss et al., 2004; Craske et al., 2012; c.f. Buss and McDoniel, 2016). Additional work has demonstrated that it is not simply poor down-regulation of negative emotion when contexts shift to positive that is evidence of emotion inflexibility, but also weaker generation of negative emotions when contexts are explicitly negative. For example, Rottenberg and colleagues have demonstrated that poor up-regulation of negative emotion during emotionally evocative films validated to elicit negative emotion is associated with onset and maintenance of depression (Rottenberg et al., 2002, 2005). Indeed, poor responsivity to negative emotional contexts, as indexed on objective, rather than self-report, emotion indices, is a consistent finding for clinical and subthreshold levels of depression (e.g., Moran et al., 2012; c.f. Bylsma et al., 2008). Moreover, Coifman and colleagues looked at discrete negative emotion generation, including sadness and anger, across several studies and found that poor up-regulation of those specific negative emotions when contextual parameters demanded them (*via* evocative films or computer simulations) was associated with poor adjustment across a variety of community samples (Coifman et al., 2016).

Although inhibition of positive emotion is much less commonly implicated in maladjustment, there is some evidence of particular instances where context-insensitive positive emotion is central in adjustment. For example, in socially stigmatizing contexts, there is evidence that positive emotions can predict worse adjustment (e.g., victims of childhood sexual abuse: Bonanno et al., 2007; bullying among school-age children: Arsenio et al., 2000). However, broadly positive emotion generation has been shown to be adaptive, regardless of context (e.g., Coifman and Bonanno, 2010). Indeed, poor generation or up-regulation of positive emotion has clearly been implicated in maladjustment and disease. For example, Papa and Bonanno (2008) demonstrated that lower positive emotions expressed during an evocative interview predicted decreased social functioning up to two years later in at-risk college students. Moreover, Harvey and colleagues found that positive emotional expression during reports of coping predicted treatment adherence in patients with chronic illness (Harvey et al., 2016). Similar findings suggest that poor generation of positive emotion in positive contexts is broadly maladaptive and associated with poor adjustment and affective disease (e.g., Moran et al., 2012; Panaite et al., 2018). It may be that positive emotions function, in part, to facilitate greater negative emotion flexibility, through processes such as down-regulation (Fredrickson et al., 2000) and adaptive behavioral choices (Nylocks et al., 2018). Indeed, these data are wholly consistent with a recent longitudinal investigation demonstrating that trait low positive emotionality broadly predicted risk across the affective disorders for up to ten years (Kendall et al., 2015).

Despite the utility of measuring spontaneous, emotional output, several limitations warrant mentioning. First, the design of emotion context-sensitivity paradigms requires careful attention and often varies based on the elicitation medium employed (e.g., film vs. picture), population of concern, and emotion(s) targeted (Rottenberg et al., 2002; Waugh et al., 2011; Coifman et al., 2016). Such methodological heterogeneity across this nascent literature may represent a vulnerability for future replication and generalizability. Moreover, such carefully controlled paradigms often aim to measure a specific emotion or valence of emotion, paired with a respective context. While such judicious consideration in design is laudable, it may also risk oversimplifying the dynamic, emotional/contextual landscape present in everyday life. For example, while existing research demonstrates a proximal utility of expressing anger under certain circumstances (e.g., peer rejection), other moderating factors, such as social status, may play an important role as to the contextual appropriateness of displaying such behavior (Van Kleef and Côté, 2007; Coifman et al., 2016). Finally, while the current methodology facilitates the measurement of spontaneous emotional output (e.g., facial coding, autonomic activity), it provides little insight into the mechanisms driving such behavior. However, this limitation also presents an opportunity for future research in helping to elucidate such underlying mechanisms. Indeed, it may be that studies attempting to dissect the underlying components of emotion flexibility might benefit most from the inclusivity of emotion context-sensitivity research design methods.

### Flexibility in Appraisal of Emotional Experience

There is a broad literature demonstrating the association between flexibility in the appraisal of emotional experience and physical and psychological health. Indeed, how individuals appraise or conceptualize emotional experiences is increasingly emphasized in contemporary models of affective disorders and in the development of new interventions for affective disease (e.g., third-wave behavioral treatments: Hayes, 2004). Flexible appraisals of emotional events and experiences are thought to be characterized by variability and complexity and this has been assessed by researchers most often using experience sampling or diary methodology, so as to capture spontaneous dynamics of emotional appraisal in daily life.

The question of emotion appraisal flexibility has been taken up by a variety of researchers operationalizing the construct in two key ways. First, research has focused on the overall complexity of reports of emotional experience by examining within an individual how reports of negative emotion relate to reports of positive emotion. Highly polarized or inflexible reporting (i.e., events or experiences are appraised as all bad or all good) has been associated with a number of maladaptive outcomes including: higher perceived and objective indicators of stress (e.g., Zautra et al., 2000, 2001, 2002), poor adjustment over time following aversive life events (e.g., Coifman et al., 2007; Dasch et al., 2010; Pitzer and Bergeman, 2014), key individual differences including personality, age, lower well-being (e.g., Rafaeli et al., 2007; Carstensen et al., 2011; Grühn et al., 2013; Brose et al., 2015), as well as psychopathology (e.g., Borderline Personality: Coifman et al., 2012; Depression: Dejonckheere et al., 2018).

In most instances, researchers have modeled the polarity of appraisals of emotional experiences by using within-person correlations or hierarchical modeling, so as to estimate how negative emotional appraisals relate to positive emotional appraisal at any given moment in time, within a participant and using the polarity score (also termed “synchrony”, “covariation,” or the “inter-affect” correlation) as an individual differences variable. However, Zautra and colleagues invested decades in examining this phenomenon, demonstrating through a variety of both experimental lab and observational field paradigms that the polarity of an individual’s appraisal of emotional events or experiences will shift depending on available resources (c.f., *The Dynamic Model of Affect*: Zautra et al., 2001). When resources are ample, typically during low perceived stress, individuals exhibit less polarized and more complex appraisals of daily events and experiences relative to their appraisals during high stress periods when resources are limited. This potential for variability within persons suggests that there are perhaps *both* individual differences in the phenomena as well as contextual variability, a point emphasized in recent research demonstrating only moderate stability over time (e.g., *r*s at 0.30 over 12 months: Dejonckheere et al., 2018). Moreover, a recent careful examination of variability in affect reporting between and within individuals demonstrated unequivocally that within-person variability deviates from between-person indices (generally operationalized as trait levels) and that the degree of deviation meaningfully differentiated individuals at high and low levels of well-being (Brose et al., 2015). Indeed, there is evidence that in groups with psychopathology, there is less potential for state level change even when stress levels are low. For example, in a sample of adults diagnosed with borderline personality (with co-morbid affective disorders such as depression, PTSD, and social anxiety) stress and affect polarity were modeled over 3 weeks of experience sampling and compared to that of healthy adults. There was a significant difference in the polarity of emotional appraisals that distinguished patients from controls during both high and low perceived stress periods, suggesting that borderline patients appraised emotional events and experiences as more polarized (i.e., inflexible) regardless of their level of perceived stress, relative to controls (Coifman et al., 2012). Importantly, there is also evidence that highly polarized emotion appraisals are predictive of maladaptive behaviors commonly associated with high-risk clinical groups, including substance use, binge eating, self-injury, and risky sexual behavior and that this association is maintained regardless of perceived stress or other contextual factors (Coifman et al., 2012).

A separate approach to research on the flexibility of emotional appraisals has been to index the moment-to-moment variability of reported negative and positive emotions over time. Although several statistical approaches have been used, one approach that relies on serial auto-correlation of appraisals is able to capture the inflexibility or rigidity of momentary reporting. High auto-correlation in negative emotion reports suggests a disconnection between appraisal of emotional experience and the typical, moderate, variability (ups and downs) of external and daily events and experiences. Indeed, Kuppens and colleagues have amassed a considerable body of research demonstrating clear associations between rigidity of negative emotional appraisals (they term this “emotional inertia”) and poor psychological health, with particularly strong associations to depression (Kuppens et al., 2010; Koval et al., 2016). Other approaches that have also considered the time-dependent nature of emotional appraisal in conjunction with psychological health include estimates also based on variability, such as the mean-squared-successive-difference, which are thought to capture instability of emotional appraisals (c.f., Ebner-Priemer et al., 2009). However, recent research has not only demonstrated the considerable overlap in these constructs but also the relative advantage of auto-correlation for detection of inflexibility in emotional appraisal, rather than the dynamics of more enduring mood-related affective processes (Koval et al., 2016).

Several limitations regarding appraisal flexibility deserve mentioning. One limitation surrounds the inconsistency in experience sampling paradigms that may contribute to heterogeneous findings (Ebner-Priemer et al., 2009). For example, the timescale used for assessments (e.g., sampling once a day vs. several times a day) may result in different portrayals of an individual’s appraisal flexibility (Koval et al., 2013). Moreover, given that the measurement of appraisal flexibility lies at the level of affect, it remains challenging to unpack whether such flexibility occurs at the level of mood or emotion, given that both mood and emotion operate on different timescales (Ekman, 1992). Another limitation surrounds the varied approach used to calculate appraisal flexibility, which can limit the ability to detect patterns of meaningful within-person variation (Zautra et al., 2000; Kuppens et al., 2010). While the various operationalizations of appraisal flexibility may share some conceptual overlap, future research may benefit from assessing the predictive utility of each simultaneously, and in relation to health outcomes, as well as more explicit exploration of time as a moderating factor (Zautra et al., 2000; Kuppens et al., 2010). This will be essential in order to begin to understand how these constructs relate to each other and to disease.

In sum, the new field of research on emotion flexibility has capitalized on rigorous measurement of real-time emotion across domains, contexts, and response modes that is less typical in conventional emotion regulation research. Indeed, a key strength of the body of emotion flexibility research is that across lines of inquiry, results are broadly consistent: emotion flexibility predicts psychological health and adjustment and emotion in-flexibility predicts affective dysfunction and maladaptive behavior consistent with affective disease. Although emotion in-flexibility research in clinical samples has typically targeted high-risk groups where emotion dysfunction is particularly evident (e.g., depression, post-traumatic stress disorder, borderline personality), that similar findings are present in stressed community samples, where presumably psychopathology is normally distributed, suggests that these findings are likely trans-diagnostic and due to underlying deficits in emotion processing circuitry.

## Emotion In-Flexibility and the Imbalance of Constituent Dimensions

There is now compounding evidence suggesting the critical importance of circuitry linking top-down control and bottom-up activation in emotion processing (LeDoux and Phelps, 2008; Ochsner et al., 2009; Hartley and Phelps, 2010; Menon, 2011). In particular, considerable neuroimaging data implicate the limbic region in bottom-up emotion activation (sometimes termed: threat sensitivity, negative affectivity, trait anxiety, neuroticism, behavioral inhibition) most notably involving the amygdale (Cunningham and Brosch, 2012). By contrast, top-down regulatory or cognitive control involves the prefrontal cortex (Kane and Engle, 2002; Braver et al., 2010; Perlman and Pelphrey, 2010) in regions consistent with traditional cognitive processing elements (i.e., attention, working memory, inhibition, Hofmann et al., 2009; Kaplan and Berman, 2010; Shackman et al., 2011; Pourtois et al., 2012). However, this classic bottom-up, top-down view has become increasingly murky. Most recent models suggest a complex interaction between amygdala activity in response to sensory input which broadly project to cortical and subcortical regions. This is in contrast to activity originating in cortical regions (e.g., dorso-lateral prefrontal cortex) that may inhibit the amygdala through its connection with the medial prefrontal cortex (LeDoux and Phelps, 2008). However, some regions of the medial prefrontal cortex (Prelimbic cortex vs. infralimbic cortex) are involved in *activating* fear versus *inhibiting* fear and both of these regions have extensive reciprocal connections with the amygdale (Pape and Pare, 2010). In sum, there is compelling evidence of complex bi-directional controls over emotion responding and the complexity of these processes may make them particularly challenging to disentangle in humans (LeDoux, 2012).

Despite this complexity, some research and theory have suggested that high bottom-up activation (i.e., threat sensitivity) combined with low top-down control resources may broadly be the source of most affective impairment, including patterns of emotion responding consistent with emotion in-flexibility. For example, recently Coifman and colleagues investigated how varying cognitive control resources impacted the flexibility of emotion responding in high-threat-sensitive adults during an ostracism simulation (Coifman et al., 2018). Although all participants were highly threat-sensitive, in-flexible emotional responding was predicted by those who also had lower levels of cognitive control (here indexed as set-shifting) during explicit rejection. In contrast, individuals high in threat sensitivity with higher cognitive control resources demonstrated greater flexibility on both autonomic and behavioral indices of emotion, generating adaptive emotional responses, including positive emotion despite the social demands. Other research has demonstrated the particular riskiness of high threat sensitivity and low cognitive control resources and tied that explicitly to high-risk symptoms of affective disease, including suicide (Venables et al., 2015). Indeed, broadly, deficits in prefrontal processing consistent with areas involved in cognitive control appear to result in prolonged negative emotion (responses are not heightened, just more enduring; Dannlowski et al., 2009) and worry (e.g., Stout et al., 2015). However, notably, there is also evidence to suggest that the reverse condition, weak bottom-up activation paired with higher levels of cognitive control, also may have considerable costs, most notably in the regulation of attention (e.g., Dennis and Chen, 2007). These data are only few of an increasing body of work attempting to both capture and explicate the complexity of these dimensions on emotion processing, given the increasing evidence of overlap with common symptoms of affective disease (Cuthbert, 2014).

## Emotion In-Flexibility and Risk: Where are we Now?

Although the foundation for understanding emotion flexibility is growing, there is a significant need for greater research attention so as to maximize the utility of this construct in understanding psychological health and risk for affective disease. Indeed, each of the current lines of inquiry has contributed broadly to an increasingly dominant view that flexibility in emotion processing is broadly healthy and that rigidity or in-flexibility is risky and may be characteristic of affective disease. However, the current work suffers from several key limitations that must be overcome in order to move the field further.

A clear weakness of the burgeoning literature on emotion inflexibility is a lack of cross-validation. Rarely do researchers investigate more than one operationalization of flexibility in a given sample and therefore it remains wholly unclear if emotion regulation flexibility is associated with apprasial flexibility or flexible context-sensitive emotion. Theoretically, all three manifestations of emotion flexibility should cohere but it may be that a particular operationalization of the construct has greater predictive utility when evaluating risk or modeling symptom development. This has yet to be explored or tested explicitly. Indeed, very little is known not just about the relations between the “types” of flexiblity, but also about the stability of these constructs over time. With two notable exceptions (Westphal et al., 2010; Dejonckheere et al., 2018), there has been little attempt to model how flexibility is maintained within an individual over months or even years. Such knowledge is essential to better understand the potential protective components of emotion flexibility as well as to better model the development of emotion in-flexibility.

One possible solution to the challenge of both cross-validation and stability is the increased collaboration of scientists across disciplines and methodological domains. A particular challenge of emotion flexibility as it has been studied is that the construct relies on diverse methodology, spanning the laboratory to the field, from the psychophysiology suite to behavioral coding. Indeed, rarely do affective scientists necessarily have methodological specialization across domains. Perhaps an increased emphasis on cross-validation or replication from lab to field would encourage greater collaboration and a more robust test of this construct, as well as some evidence of stability within and between individuals.

In addition, there is a clear need to tie specific patterns of emotion flexibility more tightly to clinical phenomena, rather than clinical populations. For example, what is the unique association between up-generation of positive emotion in positive contexts and specific symptoms of affective disorders? Arguably, poor regulation of positive emotion is one of the most clear risk factors across a constellation of affective disorders. Yet it remains unclear if poor generation of positive emotion contributes to specific symptoms more than others. Moreover, what is the relative contribution of poor generation of positive emotion as compared to poor inhibition of negative emotion in risk for disease? Patients also likely vary across these phenomena and comparing specific manifestations of emotion inflexibility within and between patient groups might better specify models of disease onset, progression, maintenance, and even relapse.

The challenge of tying a complex, largely automatic process such as emotion flexibility to specific symptoms or related clinical information is substantial. However, it is a challenge for which our field may be particularly well positioned to approach at this time. Increasingly, categorical models of disease are less emphasized whereas dimensional models are in development and their supporting research funded. Hence, meaningful clusters of symptoms that are viewed as transdiagnostic are now more clearly evident (e.g., self-defeating or impulsive behaviors: Johnson et al., 2013). In addition, a number of significant statistical innovations have crossed into the social sciences that might facilitate more efficient modeling of the complex systems that drive system development. One promising possibility is the application of advanced network models that rely on machine learning methods of estimation (Aliferis et al., 2010). In particular, these methods allow for the efficient management of larger sets of variables with levels of redundancy and interaction that are still unknown. Indeed, graph models are a key method to identify interactions across variables as they relate an outcome of interest. They can identify sets of variables that are probabilistically related to each other in complex, sometimes non-linear ways (Pearl, 2009). This approach is similar to path models but does not specify *a priori* pathways for testing; instead, significant pathways are uncovered empirically (Spirtes et al., 2000). It is possible, if not likely, that these methods will help to facilitate more truly empirically derived models of disease manifestation, within which emotion inflexibility is likely to be highly relevant.

Although this review has focused on emotional flexibility research in adult populations, it is also imperative to integrate and build upon findings from the developmental literature on emotional flexibility (or inflexibility). Indeed, one of the shortcomings of this burgeoning area of research is the relative absence of a developmental framework to inform these three lines of research on emotional inflexibility. Despite a lack of explicit integration, considerable developmental research illustrates the critical role early experience plays in shaping emotional flexibility (c.f., Coifman and Almahmoud, 2016). For example, research shows that maltreated children are at increased risk for developing aberrations in emotion attentional processing (e.g., negative emotional content bias), and this in turn, may lead to increased generation of negative emotions (Shackman et al., 2007; Shackman and Pollak, 2014). Moreover, such aberrations in emotional attentional processing may be further exacerbated, as maltreated children also show deficits in higher-order cognitive processes underlying regulatory control (Nolin and Ethier, 2007). Conversely, certain parenting styles and behaviors may also increase children’s emotion regulatory control. For example, parental modeling of positive emotion, attending to, and addressing their child’s emotions are all linked to the development of children’s complex understanding of emotions, a construct that overlaps heavily with appraisal flexibility (Denham and Kochanoff, 2002). Such emotion knowledge is fundamental to a child’s development, as it is linked with better adjustment and reduced risk (Denham et al., 2002; Ensor et al., 2011).

In addition, there is also a need for less costly methods to index emotion flexibility. Across lines of inquiry, there is consistency in findings which is a great strength and likely dependent, in part, on heavy reliance on objective indicators of emotion assessed multiple times, across contexts. However, the benefits of objective indices come with a considerable price tag, one that makes it virtually impossible to assess emotion inflexibility in an applied setting. Indeed, the least costly line of research has involved experience sampling, which still demands considerable researcher time to clean and aggregate data in order to analyze it effectively. This limitation is compounded by the increasing evidence of the weaknesses of self-report instruments. Although often a preferred cheaper alternative, self-report indices of emotion and emotion regulation demonstrate consistently poor concordance with behavioral indices and weak predictive utility (e.g., Coifman et al., 2016) as well as poor coherence across state and trait reporting (e.g., Brockman et al., 2017). However, despite this limitation, a promising new instrument indexing a component of flexibility has been developed which has thus far shown some concordance with behavioral measures (i.e., the F.R.E.E: Flexible Regulation of Emotional Expression, Burton and Bonanno, 2015). In addition, there is increasing access to low-cost or free app-based research products that can be used on any smartphone (e.g., PACO, Personal Analytics Corporation, Morris and Aguilera, 2012) that can make experience sampling research less costly and more easily accessible to non-experts. Finally, our own team as well as others are developing stand-alone assessment tools that index components of the processes that appear to underlie emotion flexibility. For example, our team has developed a brief assessment of emotion-related working memory that is free and easily applied in clinical settings. This tool can index the regulation of interference from negative emotional content and is predictive of flexible negative emotional processing both in lab and in daily life (Coifman et al., 2019). Future research will need to continue to develop, test, and refine this and other less costly alternatives.

Finally, there is need to more closely tie patterns of emotion in-flexibilty to the imblance of the constituent dimensions that underlie all emotion processing. Apart from a handful of recent studies, there has been little work tying emotion inflexibility with top-down cognitive control and bottom-up activation. For example, Myruski and colleagues adapted a task designed to assess responsivity to emotional context for use with EEG, demonstrating some association between context sensitivity and behavioral facilitation (indexed as event-related potentials) and well-being (Myruski et al., 2017). However, there is already a rapidly developing research literature of treatments attempting to address the imbalance of bottom-up versus top-down control in affective disorders (e.g., Amir et al., 2009; Britton et al., 2013; Schweizer et al., 2013). What is often missing, however, is an understanding of how these dimensions fit into the expression of emotional dysfunction in patients as well as to specific symptom constellations. Hence, there is a gap between underlying mechanisms (here the imbalance of constituent dimensions) and change in patient phenomenology, one that could be partly bridged with a greater understanding of downstream emotion processing such as the development of emotion inflexibility. Indeed, more than simply better understanding patient experience, it is increasingly clear that emotion drives behavior critical to health, adaptive and maladaptive, and patterns of emotion inflexibility have been shown to predict behaviors that maintain disease (e.g., self-injury, substance use; Coifman et al., 2012) and health (e.g., treatment adherence; Harvey et al., 2016). More work is needed to clarify these associations so as to improve the development of emotion-disease modeling as well as the effectiveness of new interventions.

## Summary and Conclusion

Broadly, theory and now burgeoning evidence suggest that flexible emotion processing, characterized by variable and responsive up- and down-regulation of emotions to meet individual needs and contextual demands, is a core feature of psychological health and adaptation. Increasingly, it is clear that patterns of emotion in-flexibility, including poor up-generation and poor down-regulation of negative and/or positive emotions, could be a phenotype for a range of affective diseases. This research has proceeded along three well-defined lines of inquiry: targeting meaningful differences in the within-person variability in emotion regulatory strategy implementation, the context-sensitivity of spontaneous emotional output, and the complexity and variability of appraisals of emotional events or experiences. These processes, like all emotion processing, are driven by a complex interplay between bottom-up threat-related neural activity and top-down cognitive control processes. There is much work still to be done to be able to maximize the construct of emotion in-flexibility as a marker for affective disease, most notably by tying patterns of in-flexibility to core symptoms of affective disorders. Some work is already underway linking emotion inflexibility to specific patient populations, but we would argue that given the trans-diagnostic features of emotion inflexibility, targeting specific symptom constellations or paths, might prove even more fruitful. Indeed, it may be that each of the three lines of research may converge to support the role of emotion in*-*flexibility in features common across affective disorders, including enduring negative mood, rumination or negative repetitive thought, and maladaptive behaviors known to maintain disease (e.g., social avoidance, substance use). In short, this developing body of work suggests a path by which future research could explicate and even exploit the ties between emotion in-flexibility and affective disease, contributing to the development of improved models of risk, assessment, and intervention, with broad implications for psychological health.

## Author Contributions

Both KC and CS developed the review ideas and wrote the manuscript.

### Conflict of Interest Statement

The authors declare that the research was conducted in the absence of any commercial or financial relationships that could be construed as a potential conflict of interest.
